# Dihalogenated trichodermin (4β-acet­oxy-9,10-dibromo-12,13-epoxy­tri­chothec)

**DOI:** 10.1107/S1600536809054178

**Published:** 2009-12-19

**Authors:** Jin-Hao Zhao, Yong Zhou, Jian-Gong Zhang, Jing-Li Cheng, Fu-Cheng Lin

**Affiliations:** a Institute of Pesticide and Environmental Toxicology, Zhejiang University, Hangzhou 310029, People’s Republic of China; b College of Chemical Engineering and Materials Science, Zhejiang University Of Technology, Hangzhou 310032, People’s Republic of China; c Institute of Biotechnology, Zhejiang University, Hangzhou 310029, People’s Republic of China

## Abstract

In the title dihalogenated trichodermin mol­ecule, C_17_H_24_Br_2_O_4_ (systematic name: 9,10-dibromo-12,13-epoxy­trichothec-9-en-4β-yl acetate), the five-membered ring displays an envelope conformation, whereas the two six-membered rings show the same conformation, *viz*. chair. As for the seven-membered ring, the dihedral angle between the mean planes formed by the four C atoms of the envelope unit and the three C and one O atoms of the six-membered chair is 69.08 (4)°; these two mean planes are nearly perpendicular to the ep­oxy ring with angles of 87.53 (4) and 88.67 (4)°, respectively.

## Related literature

For the fungicidal activity of trichodermin, see: Zhang *et al.* (2007 ▶). Trichodermin is a member of the 4β-ace­oxy-12,13-epoxy­trichothecene family, see: Nielsen *et al.* (2005 ▶). For the structure of trichodermin, see: Chen *et al.* (2008 ▶) and for the structure of a trichodermin derivative, see: Cheng *et al.* (2009 ▶).
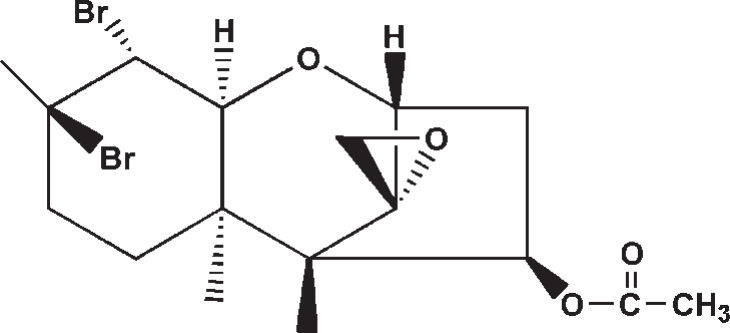


## Experimental

### 

#### Crystal data

C_17_H_24_Br_2_O_4_
*M* *_r_* = 452.18
Monoclinic, 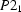

*a* = 10.0120 (4) Å
*b* = 8.3397 (4) Å
*c* = 11.1235 (6) Å
β = 106.6220 (10)°
*V* = 889.97 (7) Å^3^
*Z* = 2
Mo *K*α radiation
μ = 4.57 mm^−1^
*T* = 296 K
0.26 × 0.20 × 0.10 mm

#### Data collection

Rigaku R-AXIS RAPID diffractometer
Absorption correction: multi-scan (*ABSCOR*: Higashi, 1995 ▶) *T*_min_ = 0.323, *T*_max_ = 0.633
8698 measured reflections
3667 independent reflections
2752 reflections with *I* > 2σ(*I*)
*R*_int_ = 0.032

#### Refinement

*R*[*F* ^2^ > 2σ(*F* ^2^)] = 0.032
*wR*(*F* ^2^) = 0.095
*S* = 1.00
3667 reflections
213 parameters
1 restraint
H-atom parameters constrained
Δρ_max_ = 0.67 e Å^−3^
Δρ_min_ = −0.65 e Å^−3^
Absolute structure: Flack (1983 ▶), 1506 Friedel pairs
Flack parameter: 0.000 (15)

### 

Data collection: *PROCESS-AUTO* (Rigaku, 2006 ▶); cell refinement: *PROCESS-AUTO*; data reduction: *CrystalStructure* (Rigaku, 2007 ▶); program(s) used to solve structure: *SHELXS97* (Sheldrick, 2008 ▶); program(s) used to refine structure: *SHELXL97* (Sheldrick, 2008 ▶); molecular graphics: *ORTEP-3 for Windows* (Farrugia, 1997 ▶); software used to prepare material for publication: *WinGX* (Farrugia, 1999 ▶) and *PLATON* (Spek, 2009 ▶).

## Supplementary Material

Crystal structure: contains datablocks global, I. DOI: 10.1107/S1600536809054178/si2228sup1.cif

Structure factors: contains datablocks I. DOI: 10.1107/S1600536809054178/si2228Isup2.hkl

Additional supplementary materials:  crystallographic information; 3D view; checkCIF report

## Figures and Tables

**Fig. 1.**
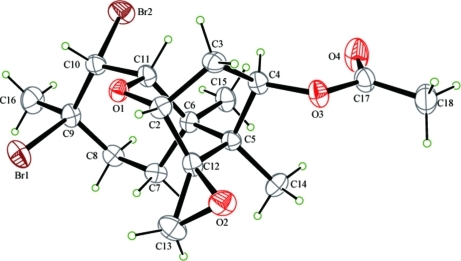
The molecular structure of (I), showing the atom-labelling scheme. Displacement ellipsoids are drawn at the 40% probability level.

## supplementary crystallographic information

### Comment

The endophytic fungi Trichoderma taxi *sp*. nov. from Taxus mairei S.Y.Hu can produce a compound with fungicidal activity – Trichodermin (Zhang *et al.*, 2007), which is a member of the 4β-aceoxy-12,13-epoxytrichothecene family (Nielsen *et al.*, 2005). Bioassays showed Trichodermin strongly inhibited Rhizoctonia solani and Botrytis cinere. In order to find the relationship between the double bond of C position and biological activities, we designed to take the halogenation reaction, thus, the title compound had been synthesized. Its molecular structure is shown in Fig. 1. In the molecule, the five membered ring displays an envelope conformation with atom C12 at the flap position 0.694 (5) Å out of the mean plane formed by C2,C3,C4,C5. The pyranyl ring displays a chair conformation with the deviations of C11 and C12 being -0.578 (5) and 0.843 (5) Å, respectively. As well as cyclopentyl ring, with C7 and C10 at the flap positions deviating by 0.685 (5) and -0.464 (5) Å, respectively. And it is interesting that the ring change to the typical chair conformation after the double bond was being displaced by Br atoms, comparing with the structure of Trichodermin (Chen *et al.*, 2008) and Trichodermol (4α-hydroxy- 12,13-epoxytrichothec-9-ene) (Cheng *et al.*, 2009). As for the seven-membered ring, the dihedral angle between the mean planes formed by C2,C3,C4,C5 and C2,C5,C6,O1 is 69.08 (4) °, which are nearly perpendicular to the epoxy ring with angles of 87.53 (4) and 88.67 (4) °, respectively.

### Experimental

In a flask, Br_2_(219 mg, 13.7 mmol, 2 equal) with 5 ml dichloromethane was added dropwise into a solution of Trichodermin (200 mg, 6.84 mmol, 1 equal), pyridine (108 mg, 13.7 mmol, 2 equal) and dichloromethane(15 ml). After stirring for 3 h at room temperature. The solution was washed with 1 N HCl, sat.NaHCO_3_ and dried over anhydrous Na_2_SO_4_. The solvent was evaporated *in vacuo* to afford the crude product, which was purified by column chromatography to afford the tittle compound (271 mg, 88%) as a white solid. The solid was filtrated and recrystallized with 95% ethanol to colourless blocks. The 1H NMR, ESI-MS data testified the title compound's structure. ESI-MS: 475.2 (*M*+Na)+ (100%); 1*H*-NMR (500 MHz, CDCl3): 5.57 (1*H*, m, H– 4), 4.62 (1*H*, s, H-10), 4.03 (1*H*, s, H-11), 3.87 (1*H*, d, J=5.0 Hz, H-2), 3.21 (1*H*, d, J=4.0 Hz, H-13), 2.94 (1*H*, d, J=4.0 Hz, H-13), 2.49–2.45 (1*H*, m, H-3), 2.42–2.36 (1*H*, m, H-8), 2.08 (3*H*, s, H-16), 2.06 (3*H*, s, H-18),2.01–1.98 (1*H*, m, H-3), 1.97–1.93 (1*H*, m, H-8), 1.92–1.88 (1*H*, m, H-7), 1.46–1.42 (1*H*, m, H-7), 1.38 (3*H*, s, H-14), 0.71 (3*H*, s, H-15).

### Refinement

The H atoms were geometrically placed (C–H = 0.93–0.98 Å) and refined as riding with *U*_iso_(H) = 1.2*U*_eq_(C) or 1.5*U*_eq_ (methyl C). The absolute structure has been determined by using Flack's x parameter refinement (Flack, 1983) and 1506 Friedel related pairs of reflections. The PLATON (Spek, 2009) structure validation programme was applied and indicated eight C atoms with chiralities R (C2), R (C4), S (C5), R (C6), R (C9), R (C10), S (C11), S (C12) for the title molecule.

### Crystal data

**Table:**

C_17_H_24_Br_2_O_4_	*F*(000) = 456
*M**_r_* = 452.18	*D*_x_ = 1.687 Mg m^−^^3^
Monoclinic, *P*2_1_	Mo *K*α radiation, λ = 0.71073 Å
Hall symbol: P 2yb	Cell parameters from 6808 reflections
*a* = 10.0120 (4) Å	θ = 3.1–27.4°
*b* = 8.3397 (4) Å	µ = 4.57 mm^−^^1^
*c* = 11.1235 (6) Å	*T* = 296 K
β = 106.622 (1)°	Chunk, colorless
*V* = 889.97 (7) Å^3^	0.26 × 0.20 × 0.10 mm
*Z* = 2	

### Data collection

**Table:**

Rigaku R-AXIS RAPID diffractometer	3667 independent reflections
Radiation source: rolling anode	2752 reflections with *I* > 2σ(*I*)
graphite	*R*_int_ = 0.032
Detector resolution: 10.00 pixels mm^-1^	θ_max_ = 27.4°, θ_min_ = 3.1°
ω scans	*h* = −12→12
Absorption correction: multi-scan (*ABSCOR*: Higashi, 1995)	*k* = −10→10
*T*_min_ = 0.323, *T*_max_ = 0.633	*l* = −14→14
8698 measured reflections	

### Refinement

**Table:**

Refinement on *F*^2^	Hydrogen site location: inferred from neighbouring sites
Least-squares matrix: full	H-atom parameters constrained
*R*[*F*^2^ > 2σ(*F*^2^)] = 0.032	*w* = 1/[σ^2^(*F*_o_^2^) + (0.0003*P*)^2^ + 1.980*P*] where *P* = (*F*_o_^2^ + 2*F*_c_^2^)/3
*wR*(*F*^2^) = 0.095	(Δ/σ)_max_ < 0.001
*S* = 1.00	Δρ_max_ = 0.67 e Å^−^^3^
3667 reflections	Δρ_min_ = −0.65 e Å^−^^3^
213 parameters	Extinction correction: *SHELXL97* (Sheldrick, 2008), Fc^*^=kFc[1+0.001xFc^2^λ^3^/sin(2θ)]^-1/4^
1 restraint	Extinction coefficient: 0.0189 (8)
Primary atom site location: structure-invariant direct methods	Absolute structure: Flack (1983), 1506 Friedel pairs
Secondary atom site location: difference Fourier map	Flack parameter: 0.000 (15)

### Special details

**Table:**

Geometry. All e.s.d.'s (except the e.s.d. in the dihedral angle between two l.s. planes) are estimated using the full covariance matrix. The cell e.s.d.'s are taken into account individually in the estimation of e.s.d.'s in distances, angles and torsion angles; correlations between e.s.d.'s in cell parameters are only used when they are defined by crystal symmetry. An approximate (isotropic) treatment of cell e.s.d.'s is used for estimating e.s.d.'s involving l.s. planes.
Refinement. Refinement of *F*^2^ against ALL reflections. The weighted *R*-factor *wR* and goodness of fit *S* are based on *F*^2^, conventional *R*-factors *R* are based on *F*, with *F* set to zero for negative *F*^2^. The threshold expression of *F*^2^ > σ(*F*^2^) is used only for calculating *R*-factors(gt) *etc*. and is not relevant to the choice of reflections for refinement. *R*-factors based on *F*^2^ are statistically about twice as large as those based on *F*, and *R*- factors based on ALL data will be even larger.

### Fractional atomic coordinates and isotropic or equivalent isotropic displacement parameters (Å^2^)

**Table:**

	*x*	*y*	*z*	*U*_iso_*/*U*_eq_	
Br2	0.88801 (4)	0.21090 (8)	0.71835 (5)	0.06304 (15)	
Br1	0.75860 (5)	−0.28303 (7)	0.84118 (5)	0.05950 (14)	
O2	0.2048 (3)	−0.0632 (4)	0.6936 (3)	0.0520 (9)	
O1	0.5492 (3)	−0.0758 (3)	0.6377 (3)	0.0377 (7)	
O3	0.2174 (3)	0.2951 (4)	0.5751 (3)	0.0459 (9)	
C11	0.6197 (4)	0.0702 (5)	0.6879 (5)	0.0376 (10)	
H11	0.6071	0.1471	0.6189	0.045*	
O4	0.3167 (4)	0.5380 (4)	0.6153 (5)	0.0713 (13)	
C7	0.6122 (5)	0.0479 (6)	0.9123 (4)	0.0408 (11)	
H7A	0.5826	0.1011	0.9779	0.049*	
H7B	0.5681	−0.0567	0.8986	0.049*	
C6	0.5623 (4)	0.1472 (5)	0.7900 (4)	0.0353 (10)	
C12	0.3494 (4)	−0.0260 (5)	0.7073 (4)	0.0378 (10)	
C2	0.3997 (4)	−0.0627 (5)	0.5960 (4)	0.0351 (10)	
H2	0.3575	−0.1619	0.5549	0.042*	
C5	0.3968 (4)	0.1468 (5)	0.7378 (4)	0.0344 (9)	
C13	0.3133 (5)	−0.1439 (7)	0.7910 (5)	0.0545 (14)	
H13A	0.3312	−0.2561	0.7786	0.065*	
H13B	0.3253	−0.1129	0.8775	0.065*	
C15	0.6110 (5)	0.3216 (6)	0.8173 (5)	0.0486 (14)	
H15A	0.7107	0.3243	0.8497	0.073*	
H15B	0.5702	0.3664	0.8781	0.073*	
H15C	0.5825	0.3831	0.7413	0.073*	
C8	0.7705 (5)	0.0260 (6)	0.9561 (4)	0.0430 (12)	
H8A	0.8141	0.1303	0.9755	0.052*	
H8B	0.7950	−0.0367	1.0328	0.052*	
C17	0.2139 (5)	0.4560 (6)	0.5912 (5)	0.0469 (12)	
C10	0.7743 (5)	0.0191 (6)	0.7299 (5)	0.0419 (11)	
H10	0.7879	−0.0600	0.6693	0.050*	
C18	0.0687 (6)	0.5157 (7)	0.5733 (6)	0.0670 (17)	
H18A	0.0335	0.4743	0.6388	0.100*	
H18B	0.0103	0.4804	0.4934	0.100*	
H18C	0.0692	0.6307	0.5762	0.100*	
C4	0.3545 (4)	0.2220 (7)	0.6039 (4)	0.0385 (9)	
H4	0.4240	0.3011	0.5963	0.046*	
C14	0.3263 (4)	0.2247 (8)	0.8282 (4)	0.0471 (10)	
H14A	0.2275	0.2070	0.7987	0.071*	
H14B	0.3448	0.3378	0.8327	0.071*	
H14C	0.3623	0.1781	0.9100	0.071*	
C9	0.8282 (5)	−0.0555 (6)	0.8612 (5)	0.0431 (12)	
C3	0.3483 (5)	0.0813 (5)	0.5121 (4)	0.0401 (11)	
H3A	0.2538	0.0644	0.4596	0.048*	
H3B	0.4080	0.1018	0.4588	0.048*	
C16	0.9878 (5)	−0.0734 (8)	0.9074 (6)	0.0654 (17)	
H16A	1.0299	0.0307	0.9240	0.098*	
H16B	1.0199	−0.1260	0.8441	0.098*	
H16C	1.0130	−0.1361	0.9829	0.098*	

### Atomic displacement parameters (Å^2^)

**Table:**

	*U*^11^	*U*^22^	*U*^33^	*U*^12^	*U*^13^	*U*^23^
Br2	0.0445 (2)	0.0665 (3)	0.0795 (3)	−0.0133 (4)	0.0199 (2)	0.0144 (4)
Br1	0.0729 (3)	0.0375 (2)	0.0607 (3)	0.0006 (4)	0.0071 (2)	0.0039 (3)
O2	0.0365 (16)	0.058 (2)	0.060 (2)	−0.0100 (17)	0.0112 (15)	0.0042 (18)
O1	0.0380 (15)	0.0318 (16)	0.0433 (17)	−0.0010 (14)	0.0116 (12)	−0.0068 (13)
O3	0.0347 (16)	0.0360 (17)	0.061 (2)	0.0020 (14)	0.0032 (15)	0.0043 (15)
C11	0.032 (2)	0.030 (2)	0.049 (3)	−0.0038 (19)	0.0086 (19)	0.0006 (19)
O4	0.058 (2)	0.036 (2)	0.118 (4)	−0.0057 (19)	0.021 (2)	−0.010 (2)
C7	0.038 (2)	0.047 (3)	0.036 (2)	−0.006 (2)	0.0077 (18)	0.0024 (19)
C6	0.036 (2)	0.031 (2)	0.038 (2)	−0.0035 (18)	0.0074 (17)	−0.0069 (18)
C12	0.0303 (19)	0.031 (2)	0.049 (3)	−0.0030 (19)	0.0062 (18)	0.0009 (19)
C2	0.036 (2)	0.034 (2)	0.034 (2)	−0.007 (2)	0.0081 (17)	−0.0089 (18)
C5	0.0346 (19)	0.030 (2)	0.038 (2)	−0.0037 (18)	0.0105 (17)	0.0000 (17)
C13	0.056 (3)	0.047 (3)	0.054 (3)	−0.014 (3)	0.006 (2)	0.007 (2)
C15	0.048 (3)	0.033 (2)	0.062 (3)	−0.005 (2)	0.010 (2)	−0.006 (2)
C8	0.040 (2)	0.043 (3)	0.041 (3)	−0.005 (2)	0.0032 (19)	0.000 (2)
C17	0.045 (3)	0.034 (2)	0.060 (3)	0.003 (2)	0.014 (2)	0.004 (2)
C10	0.037 (2)	0.038 (2)	0.052 (3)	−0.004 (2)	0.016 (2)	0.005 (2)
C18	0.052 (3)	0.053 (3)	0.097 (5)	0.014 (3)	0.024 (3)	0.012 (3)
C4	0.0332 (17)	0.033 (2)	0.047 (2)	−0.003 (3)	0.0073 (15)	0.001 (3)
C14	0.0443 (19)	0.052 (3)	0.049 (2)	0.001 (3)	0.0197 (17)	−0.012 (3)
C9	0.032 (2)	0.042 (3)	0.049 (3)	0.000 (2)	0.0023 (19)	−0.001 (2)
C3	0.046 (2)	0.043 (3)	0.030 (2)	−0.001 (2)	0.0093 (19)	−0.0010 (18)
C16	0.040 (3)	0.075 (4)	0.075 (4)	0.008 (3)	0.005 (3)	0.005 (3)

### Geometric parameters (Å, °)

**Table:**

Br2—C10	1.989 (5)	C13—H13A	0.9700
Br1—C9	2.012 (5)	C13—H13B	0.9700
O2—C12	1.445 (5)	C15—H15A	0.9600
O2—C13	1.461 (6)	C15—H15B	0.9600
O1—C11	1.438 (5)	C15—H15C	0.9600
O1—C2	1.439 (5)	C8—C9	1.503 (7)
O3—C17	1.355 (6)	C8—H8A	0.9700
O3—C4	1.451 (5)	C8—H8B	0.9700
C11—C10	1.544 (6)	C17—C18	1.494 (7)
C11—C6	1.551 (7)	C10—C9	1.536 (7)
C11—H11	0.9800	C10—H10	0.9800
O4—C17	1.200 (6)	C18—H18A	0.9600
C7—C8	1.530 (6)	C18—H18B	0.9600
C7—C6	1.548 (6)	C18—H18C	0.9600
C7—H7A	0.9700	C4—C3	1.546 (7)
C7—H7B	0.9700	C4—H4	0.9800
C6—C15	1.536 (6)	C14—H14A	0.9600
C6—C5	1.592 (6)	C14—H14B	0.9600
C12—C13	1.469 (7)	C14—H14C	0.9600
C12—C2	1.496 (6)	C9—C16	1.539 (6)
C12—C5	1.524 (6)	C3—H3A	0.9700
C2—C3	1.518 (6)	C3—H3B	0.9700
C2—H2	0.9800	C16—H16A	0.9600
C5—C14	1.529 (6)	C16—H16B	0.9600
C5—C4	1.558 (6)	C16—H16C	0.9600
			
C12—O2—C13	60.7 (3)	C9—C8—C7	113.7 (4)
C11—O1—C2	114.3 (3)	C9—C8—H8A	108.8
C17—O3—C4	116.4 (4)	C7—C8—H8A	108.8
O1—C11—C10	102.8 (3)	C9—C8—H8B	108.8
O1—C11—C6	113.1 (3)	C7—C8—H8B	108.8
C10—C11—C6	116.2 (4)	H8A—C8—H8B	107.7
O1—C11—H11	108.1	O4—C17—O3	122.7 (4)
C10—C11—H11	108.1	O4—C17—C18	125.4 (5)
C6—C11—H11	108.1	O3—C17—C18	111.9 (4)
C8—C7—C6	112.9 (4)	C9—C10—C11	116.7 (4)
C8—C7—H7A	109.0	C9—C10—Br2	109.6 (3)
C6—C7—H7A	109.0	C11—C10—Br2	107.5 (3)
C8—C7—H7B	109.0	C9—C10—H10	107.6
C6—C7—H7B	109.0	C11—C10—H10	107.6
H7A—C7—H7B	107.8	Br2—C10—H10	107.6
C15—C6—C7	109.1 (4)	C17—C18—H18A	109.5
C15—C6—C11	112.0 (4)	C17—C18—H18B	109.5
C7—C6—C11	109.2 (4)	H18A—C18—H18B	109.5
C15—C6—C5	108.2 (3)	C17—C18—H18C	109.5
C7—C6—C5	111.1 (4)	H18A—C18—H18C	109.5
C11—C6—C5	107.3 (3)	H18B—C18—H18C	109.5
O2—C12—C13	60.2 (3)	O3—C4—C3	108.5 (3)
O2—C12—C2	115.7 (4)	O3—C4—C5	111.2 (3)
C13—C12—C2	126.1 (4)	C3—C4—C5	105.9 (4)
O2—C12—C5	117.7 (4)	O3—C4—H4	110.4
C13—C12—C5	127.5 (4)	C3—C4—H4	110.4
C2—C12—C5	102.8 (4)	C5—C4—H4	110.4
O1—C2—C12	108.3 (3)	C5—C14—H14A	109.5
O1—C2—C3	113.3 (4)	C5—C14—H14B	109.5
C12—C2—C3	102.2 (4)	H14A—C14—H14B	109.5
O1—C2—H2	110.9	C5—C14—H14C	109.5
C12—C2—H2	110.9	H14A—C14—H14C	109.5
C3—C2—H2	110.9	H14B—C14—H14C	109.5
C12—C5—C14	112.0 (4)	C8—C9—C10	112.6 (4)
C12—C5—C4	100.7 (4)	C8—C9—C16	112.4 (4)
C14—C5—C4	114.0 (4)	C10—C9—C16	113.9 (4)
C12—C5—C6	108.0 (3)	C8—C9—Br1	108.3 (3)
C14—C5—C6	112.8 (3)	C10—C9—Br1	105.1 (3)
C4—C5—C6	108.5 (3)	C16—C9—Br1	103.8 (3)
O2—C13—C12	59.1 (3)	C2—C3—C4	104.6 (4)
O2—C13—H13A	117.9	C2—C3—H3A	110.8
C12—C13—H13A	117.9	C4—C3—H3A	110.8
O2—C13—H13B	117.9	C2—C3—H3B	110.8
C12—C13—H13B	117.9	C4—C3—H3B	110.8
H13A—C13—H13B	115.0	H3A—C3—H3B	108.9
C6—C15—H15A	109.5	C9—C16—H16A	109.5
C6—C15—H15B	109.5	C9—C16—H16B	109.5
H15A—C15—H15B	109.5	H16A—C16—H16B	109.5
C6—C15—H15C	109.5	C9—C16—H16C	109.5
H15A—C15—H15C	109.5	H16A—C16—H16C	109.5
H15B—C15—H15C	109.5	H16B—C16—H16C	109.5
			
C2—O1—C11—C10	177.3 (4)	C11—C6—C5—C14	−178.3 (4)
C2—O1—C11—C6	51.2 (5)	C15—C6—C5—C4	70.1 (5)
C8—C7—C6—C15	−68.6 (5)	C7—C6—C5—C4	−170.1 (4)
C8—C7—C6—C11	54.1 (5)	C11—C6—C5—C4	−50.9 (5)
C8—C7—C6—C5	172.2 (4)	C2—C12—C13—O2	−101.5 (5)
O1—C11—C6—C15	−165.1 (3)	C5—C12—C13—O2	103.6 (5)
C10—C11—C6—C15	76.2 (5)	C6—C7—C8—C9	−58.6 (5)
O1—C11—C6—C7	73.9 (4)	C4—O3—C17—O4	7.5 (7)
C10—C11—C6—C7	−44.7 (5)	C4—O3—C17—C18	−173.4 (4)
O1—C11—C6—C5	−46.6 (5)	O1—C11—C10—C9	−85.2 (5)
C10—C11—C6—C5	−165.2 (4)	C6—C11—C10—C9	38.9 (6)
C13—O2—C12—C2	118.6 (5)	O1—C11—C10—Br2	151.3 (3)
C13—O2—C12—C5	−119.5 (5)	C6—C11—C10—Br2	−84.6 (4)
C11—O1—C2—C12	−63.9 (5)	C17—O3—C4—C3	−145.6 (4)
C11—O1—C2—C3	48.7 (5)	C17—O3—C4—C5	98.4 (5)
O2—C12—C2—O1	−159.2 (3)	C12—C5—C4—O3	94.1 (4)
C13—C12—C2—O1	−88.6 (5)	C14—C5—C4—O3	−26.0 (6)
C5—C12—C2—O1	71.2 (4)	C6—C5—C4—O3	−152.6 (4)
O2—C12—C2—C3	80.9 (4)	C12—C5—C4—C3	−23.5 (4)
C13—C12—C2—C3	151.5 (4)	C14—C5—C4—C3	−143.6 (4)
C5—C12—C2—C3	−48.7 (4)	C6—C5—C4—C3	89.8 (4)
O2—C12—C5—C14	37.6 (5)	C7—C8—C9—C10	48.9 (5)
C13—C12—C5—C14	−34.7 (6)	C7—C8—C9—C16	179.1 (4)
C2—C12—C5—C14	165.9 (3)	C7—C8—C9—Br1	−66.8 (4)
O2—C12—C5—C4	−84.0 (4)	C11—C10—C9—C8	−39.4 (6)
C13—C12—C5—C4	−156.2 (4)	Br2—C10—C9—C8	83.1 (4)
C2—C12—C5—C4	44.3 (4)	C11—C10—C9—C16	−168.8 (4)
O2—C12—C5—C6	162.4 (4)	Br2—C10—C9—C16	−46.3 (5)
C13—C12—C5—C6	90.1 (5)	C11—C10—C9—Br1	78.3 (4)
C2—C12—C5—C6	−69.3 (4)	Br2—C10—C9—Br1	−159.3 (2)
C15—C6—C5—C12	178.4 (4)	O1—C2—C3—C4	−83.8 (4)
C7—C6—C5—C12	−61.8 (5)	C12—C2—C3—C4	32.6 (4)
C11—C6—C5—C12	57.4 (4)	O3—C4—C3—C2	−124.6 (4)
C15—C6—C5—C14	−57.3 (5)	C5—C4—C3—C2	−5.1 (4)
C7—C6—C5—C14	62.5 (5)		
